# *Bixa orellana* leaf extract suppresses histamine-induced endothelial hyperpermeability via the PLC-NO-cGMP signaling cascade

**DOI:** 10.1186/s12906-015-0901-3

**Published:** 2015-10-14

**Authors:** Yoke Keong Yong, Hoe Siong Chiong, Muhd Nazrul Somchit, Zuraini Ahmad

**Affiliations:** Department of Human Anatomy, Faculty of Medicine and Health Sciences, Universiti Putra Malaysia, 43400 Serdang, Selangor Malaysia; Department of Biomedical Science, Faculty of Medicine and Health Sciences, Universiti Putra Malaysia, 43400 Serdang, Selangor Malaysia

**Keywords:** *Bixa Orellana*, Histamine, Endothelial permeability

## Abstract

**Background:**

Histamine is established as a potent inflammatory mediator and it is known to increased endothelial permeability by promoting gap formation between endothelial cells. Previous studies have shown that aqueous extract of *Bixa orellana* leaves (AEBO) exhibits antihistamine activity *in vivo*, yet the mechanism of its action on endothelial barrier function remains unclear. Therefore, the current study aimed to determine the protective effect of AEBO against histamine-induced hyperpermeability *in vitro*.

**Methods:**

The endothelial protective effect of AEBO was assess using an *in vitro* vascular permeability assay kit. Human umbilical vein endothelial cells (HUVEC) were used in the current study. HUVEC were pre-treated with AEBO for 12 h before histamine induction. Vascular permeability was evaluated by the amount of FITC-dextran leakage into the lower chamber. In order to elucidate the mechanism of action of AEBO, phospholipase C (PLC) activity, intracellular calcium level, nitric oxide (NO) concentration, cyclic guanosine monophosphate (cGMP) production and protein kinase C (PKC) activity were determined following histamine challenge.

**Results:**

Histamine-induced increased HUVEC permeability was significantly attenuated by pretreatment with AEBO in a time- and concentration-dependent manner. Upregulation of PLC activity caused by histamine in HUVEC was suppressed by pretreatment with AEBO. Pretreatment with AEBO also blocked the production of intracellular calcium induced by histamine in HUVEC. In addition, AEBO suppressed the NO-cGMP signaling cascade when HUVEC were challenged with histamine. Moreover, PKC activity was significantly abolished by pretreatment with AEBO in HUVEC under histamine condition.

**Conclusion:**

In conclusion, the present data suggest that AEBO could suppress histamine-induced increased endothelial permeability and the activity may be closely related with the inhibition of the PLC-NO-cGMP signaling pathway and PKC activity.

## Background

Vascular endothelium plays a critical role as a sophisticated gate-keeper that regulates the passage of solutes, macromolecules and circulating cells across the blood vessel wall [1]. Thus, endothelial permeability is important for the maintenance of vascular integrity in either homeostasis or disease. However, disintegration of endothelial cell junctions will lead to increased endothelial permeability and subsequently, the formation of edema. Endothelial hyperpermeaility is a hallmark of many severe disorders, such as ischemic acute renal failure [2] and sepsis [3]. Various factors contribute to endothelial hyperpermeability, for example, inflammatory mediators, oxidants and cytokines.

Histamine, produced by mast cells and macrophages [4], is a known potent inflammatory mediator. By binding to its H_1_ receptor on endothelial cells, histamine can cause the formation of gaps between endothelial cells, eventually leading to endothelial hyperpermeability [5]. These effects involve multiple signalling cascades, for instance the activation of protein kinase C [6], the generation of inositol trisphosphate and a rise in intracellular Ca^2+^ [7]. In addition, histamine-stimulated endothelial hyperpermeability also involves the nitric oxide—cyclic guanosine monophosphate (NO-cGMP) signaling pathway [8]. Despite active research, treatment for endothelial dysfunction remains widely lacking; this opens an interest among researchers to explore new therapeutic options for the prevention of endothelial leakage.

*Bixa orellana*, also known as “annatto”, is famous for its colorant properties as well as its medicinal value. Apart from antioxidant, antibacterial, analgesic [9], antileishmanial and antifungal activities [10], leaves of *B. orellana* have also been documented to have anti-inflammatory activity [11]. Previous studies have also shown that aqueous extracts of *B. orellana* leaves possessed antihistamine activity [12], and more recently, Yoke Keong and colleagues (2013) have demonstrated that *B. orellana* leaves are capable of suppressing endothelial hyperpermeability stimulated by serotonin *in vivo* [13]. However, the mechanism of action of *B. orellana* leaf extract remains unclear. Therefore, the aim of this study was to explore the protective effect of *B. orellana* against histamine-induced hyperpermebility in human umbilical vein endothelial cells (HUVEC). When a positive response was demonstrated, the effects of the extract on signaling pathways that regulate endothelial permeability were subsequently studied. Experimental data suggested that AEBO protected the endothelial cell barrier against histamine disruption by suppressing the phospholipase C (PLC)–NO–cGMP signaling pathway.

## Methods

### Plant material

*B. orellana* leaves were collected from around Universiti Putra Malaysia and their botanical identity was identified and confirmed by the Phytomedicinal Herbarium, Institute of Biosciences, Universiti Putra Malaysia, Selangor, Malaysia with the voucher specimen, No. NL16, *Bixa orellana*. Leaves were then washed and dried at 60 °C in an oven and powdered.

### Preparation of plant extract

The plant extraction method was followed as reported in a previous study [11]. Briefly, powdered leaves were soaked in distilled water (1 g powder:20 ml distilled water), and incubated in a water bath at 40 °C for 24 h. The mixture was filtered and the filtrate was then freeze-dried, yielding an aqueous extract (8.5 %. w/w).

### Drugs and chemicals

Histamine, loratadine, fura-2-acetoxymethyl ester (FURA-2 AM), verapamil, 2-aminoethoxydiphenyl borate (2-APB), ethylene glycol tetraacetic acid (EGTA), Hank’s Balanced Salt solution (HBSS), phosphate buffered saline (PBS) and 10× trypsin-EDTA solution were purchased from Signal Chemical Co. Ltd. Malaysia. 1-[6-[[(17β)-3-methoxyestra-1,3,5(10)-trien-17-yl]amino]hexyl]-1H-pyrrole-2,5-dione (U-73122), L-N^G^-Nitroarginine methyl ester hydrochloride (L-NAME), 6-(phenylamino)-5,8-quinolinedione (LY 83583) and GF 109203X hydrochloride were purchased from Merck, Malaysia.

### Cell culture

HUVEC and growth medium (M200) supplemented with a low-serum growth supplement were purchased from Cascade Biologics (Portland). Cells were maintained at 37 °C in 5 % CO_2_ and 95 % ambient air in a humidified cell culture incubator. Only cells from passages 1 to 5 were used in experiments. Cells were used for experiments once they reached > 90 % confluence.

### *In vitro* vascular permeability assay

The trans-endothelial flux of FITC-dextran across cultured endothelial cell monolayers was measured using a commercial *in vitro* vascular permeability assay kit (Chemicon, International). Assay procedures were slightly modified from the manufacturer’s protocol. Briefly, cells were grown to confluence on collagen-coated inserts. Cells were pre-treated with AEBO at a concentration of 0.1–0.4 mg/ml for 12 h and then challenged with histamine (100 μM), followed by addition of FITC-dextran. The plate was incubated for 5 min, and to stop the reaction, the inserts were moved to another well for subsequent time-point reading (15 and 30 min). Fluorescent emissions were measured using a spectroflurometer (Tecan) with *a* 485 and 530 nm filter set (Ex/Em). Loratadine, an anti-histamine drug was used as a positive control.

The permeability index (%) was calculated as:

$$ \left[\left(\mathrm{experimental}\ \mathrm{clearance}\right)\ \hbox{--}\ \left(\mathrm{spontaneous}\ \mathrm{clearance}\right)\right]\ /\ \left[\left(\mathrm{clearance}\ \mathrm{of}\ \mathrm{the}\ \mathrm{filter}\ \mathrm{alone}\right)\ \hbox{--}\ \mathrm{spontaneous}\ \mathrm{clearance}\right)\Big]\ \mathrm{x}\ 100 $$

### Determination of Phospholipase C (PLC) activity

PLC activity was determined by quantification of inositol phosphate using a commercial IP-One ELISA kit (Cisbio, USA) and the assay procedures were modified from the manufacturer’s protocol. Cells were grown on 24-well plates and pretreated with AEBO at various concentrations for 12 h before histamine was added. 1 h after exposure to histamine, cells were lysed and centrifuged. Supernatants were transferred to a coated ELISA plate and IP1-HRP conjugate and anti-IP1 monoclonal antibodies were added. The plate was incubated for another 3 h, washed, and the reaction stopped by addition of stop solution. The plate was read at 450 nm with a wavelength correction at 620 nm using a spectrophotometer (Tecan Infinite 200). The concentration of inositol phosphate was calculated from the standard curve, which was run together with the samples. The PLC inhibitor, U-73122 was used as a positive control.

### Calcium signalling assay

Levels of intracellular calcium were measured in HUVEC monolayers by Fura-2 AM ester modifed from Grynkiewicz [14] following Chandra and Angle [15]. Briefly, cells were grown to confluence and pretreated with AEBO at various concentrations. Cells were then harvested with trypsin and washed with physiological calcium-free HBSS, then centrifuged at 1000 rpm for 5 min. Next, cell pellets were incubated with Fura-1 AM at a concentration of 2 μg/ml for 1 h at room temperature covered with aluminium foil. Cells were then washed with physiological calcium-free HBSS. Aliquots of the Fura-2 AM-loaded cell suspension at a concentration of 1 × 10^6^ cells/ml were transferred to glass cuvettes and stimulated with histamine only after 1 min of baseline recording. Readings from dual excitation at 340/380 nm with an emission wavelength of 510 nm were recorded using a Perkin-Elmer Fluorescence Spectrometer LS-55. Experiments were calibrated by the addition of 2 % (w/v) Triton X-100, 5.2 mM MnCl_2_.4H_2_O and 0.5 M EGTA. Changes in the level of intracellular calcium ([Ca^2+^]_i_) were calculated by the calibration ratio using the following equation:

$$ {\left[\mathrm{C}{\mathrm{a}}^{2+}\right]}_{\mathrm{I}} = \mathrm{k}\ \mathrm{X}\ \left(\mathrm{R}\ \hbox{--}\ {\mathrm{R}}_{\min}\right)/\ \left({\mathrm{R}}_{\max}\hbox{--}\ \mathrm{R}\right) $$

Where R is the fluorescent measurement ratio (340/380), R_max_ is the ratio when the dye is saturated with calcium obtained with 10 mM EGTA, R_min_ is the ratio with no free calcium present and k represents the product of the effective dissociation constant for Fura-2 AM and can be estimated from the calibration curve for Fura-2 AM.

### NO assay

Measurement of nitrite and nitrate as an indicator of nitric oxide production was carried out using a nitrite/nitrate assay kit (Roche, Malaysia). Briefly, cells were pre-treated with AEBO before being challenged with histamine. Samples were mixed with an equal volume of Griess reagent. The nitrate in the samples was converted to nitrite by adding nitrate reductase prior to Griess reagent. L-NAME, an endothelial nitric oxide synthase inhibitor was used as a positive control.

### Determination of cyclic Guanosine Monophosphate (cGMP) Production

Endothelial cGMP levels were quantified using a cGMP enzyme immunoassay (R & D System, USA) according to the manufacturer’s instructions. Cell culture supernatants were collected after cells were preincubated with AEBO and then induced with histamine. Supernatants were transferred onto an ELISA plate. The reaction was stopped and measured by spectrophotometry at 450 nm with a wavelength correction 570 nm within 30 min. The mean absorbance was used to calculate cGMP concentration from the standard curve.

### Quantification of Protein Kinase C (PKC) activity

PKC activity was determined using a commercial ELISA kit (Enzo Life Sciences, Malaysia). Briefly, cell lysates were prepared after 12 h of pretreatment with AEBO and 30 min of stimulation with histamine. Cell lysates were then transferred to an ELISA plate for the enzymatic reaction. The absorbance of the mixture in the plate was then measured at 450 nm using a spectrophotometer (Tecan, Infinite 200).

### Statistical analysis

All values in the figures and test results are expressed as mean ± SE. All experiments were performed in triplicate with three independent tests. Statistical analysis of data was performed by one-way analysis of variance (ANOVA) and further analysed using Dunnet’s test. *P* values less than 0.05 (*p* < 0.05) were considered significant.

## Results

### AEBO suppressed histamine-induced increased endothelial permeability

**Fig. 1:**
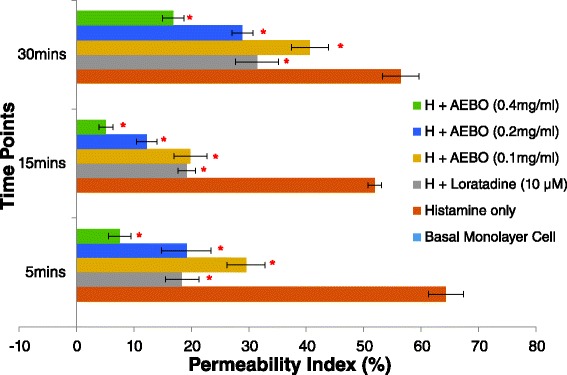
Effect of AEBO on histamine-induced increased HUVEC permeability for three different time points. Three independent experiments were performed in triplicate. Data were expressed in mean ± SEM. *P < 0.05 considered significant versus control (histamine only) in each time point

In the current study, permeability of the HUVEC monolayer was measured three times at different time points (i.e., 5, 15 and 30 min). Exposure of the monolayer to histamine significantly increased HUVEC permeability by approximately 5 to 6 fold, compared with baseline (64.31 ± 3.08, 51.94 ± 1.17 and 56.44 ± 3.16 permeability index of baseline at 5, 15 and 30 min, respectively; Fig. 1). However, pre-incubation with AEBO at 0.1 and 0.2 mg/ml significantly reduced HUVEC hyperpermeability caused by histamine at all time points and the highest inhibitory effect was exhibited at the 15^th^ min (i.e., 61.80 and 76.43 %, respectively). AEBO at 0.4 mg/ml demonstrated the highest activity among all treatment groups (88.34, 90.22 and 70.18 % at 5, 15 and 30 min, respectively). The reference drug, Loratadine also showed a significant reduction against histamine-induced increased endothelial permeability. AEBO alone did not cause increased endothelial permeability (data not shown), therefore, we concluded that this protective effect was specific to injured endothelial cells.

### AEBO attenuated PLC activity

**Fig. 2:**
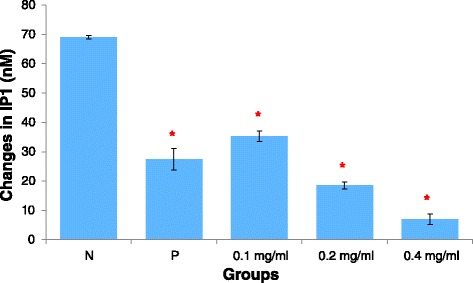
Effects of AEBO on histamine-induced production of inositol phosphate in cultured HUVEC. Three independent experiments were performed in triplicate. Data were expressed in mean ± SEM. Data were analyzed by One-way ANOVA. *P < 0.05 (N = Treated with histamine only; P = Drug control, 10 μM of U-73122)

Quantification of inositol phosphate was used to indicate PLC activity in the present study. As shown in Fig. 2, stimulation of histamine led to a rise in the inositol phosphate level, to approximately 70 ± 0.63 nM higher than the basal level. Pre-treatment with AEBO led to significant (*p* < 0.05) reductions in inositol phosphate production in HUVEC in a dose-dependent manner. Interestingly, a concentration of 0.4 mg/ml showed maximal inhibition, with a 62.14 ± 1.82 nM reduction of inositol phosphate production compared with the negative control (90.01 % inhibition). To confirm whether PLC signalling was involved in the regulation of endothelial hyperpermeability, the effect of the PLC inhibitor, U-73122 was measured. Pretreatment of HUVEC with U-73122 also successfully suppressed histamine-induced inositol phosphate production by approximately 41 ± 2.86 nM (60 % inhibition) compared with the negative control. This result suggested the involvement of PLC signalling in the regulation of histamine-induced increased endothelial permeability.

### AEBO reduced histamine-induced calcium signalling

#### Inhibition of total calcium influx

**Fig. 3:**
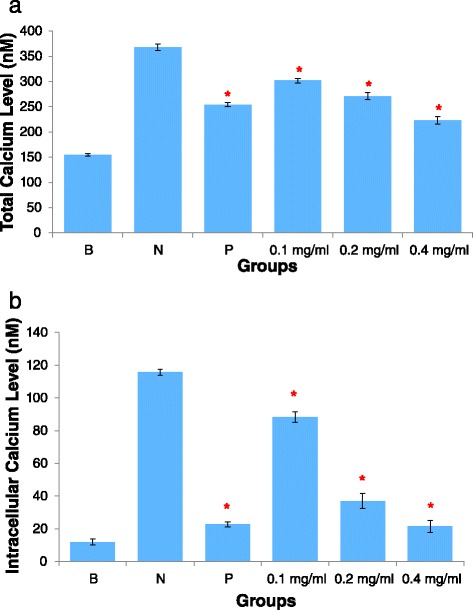
Effects of AEBO on histamine-induced [Ca^2+^]_i_ in cultured HUVEC. Total calcium level was shown in 3(**a**), while intracellular calcium level shown in 3(**b**). Three independent experiments were performed in triplicate. Data were expressed in mean ± SEM. Data were analyzed by One-way ANOVA. *P < 0.05 significant different from negative control. [B = basal level; N = Treated with histamine only; P = Drug control, 10 μM of verapamil(3a) and 2-APB(3b)]

In order to measure total calcium from both intracellular calcium release and extracellular calcium influx, HBSS containing physiological calcium levels was used. Total [Ca^2+^]_i_ increased when HUVEC was induced with histamine (Fig. 3a). However, HUVEC pre-incubated with AEBO showed a reduction in total [Ca^2+^]_i_. AEBO at 0.1 and 0.2 mg/ml suppressed total calcium influx by up to 30.95 and 45.29 %, respectively. In addition, 0.4 mg/ml of AEBO possessed the highest inhibition activity with approximately 67.94 %. The calcium channel blocker, Verapamil, when used as a reference, showed significant suppression of total [Ca^2+^]_i_.

### Inhibition of intracellular calcium release

Intracellular calcium release was measured using the same method as above, only the HBSS used was physiologically calcium free. As shown in Fig. 3b, exposure to histamine induced a massive amount of [Ca^2+^]_i_ release from HUVEC. Pre-incubation of the HUVEC monolayer with 0.1 and 0.2 mg/ml AEBO caused histamine to induce the release of 76.28 ± 2.33 nM and 24.98 ± 3.65 nM of [Ca^2+^]_i_, respectively, while in the negative control, 103.75 ± 0.78 nM Ca^2+^ was released. The maximum percentage of inhibition (90.80 %) was produced by the highest dose of AEBO (0.4 mg/ml). This showed that the effect of 0.4 mg/ml of AEBO was comparable to that of the reference drug, 2-APB, which gave 89.63 % inhibition. Overall, our data suggested that the effect of AEBO on histamine-induced endothelial hyperpermeability was, at least in part, mediated by the suppression of intracellular calcium release.

### AEBO decreased histamine-induced NO production

**Fig. 4:**
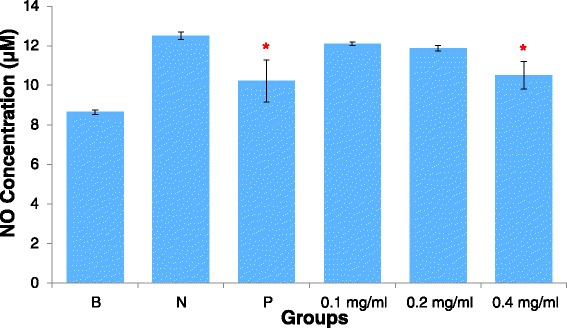
Evaluation of NO production by HUVEC stimulated with histamine which pre-treated by AEBO. Three independent experiments were performed in triplicate. Data were expressed in mean ± SEM. *P < 0.05, significant different compared with the negative control by One-way ANOVA, Post Hoc, Tukey test. (B = basal level; N = Treated with histamine only; P = Drug control, 10 μM of L-NAME)

NO production was increased by histamine (100 μM) from 8.65 ± 0.67 μM to 12.51 ± 0.88 μM, but was not significantly suppressed by any concentration of AEBO (Fig. 4). Only the highest dose of AEBO, 0.4 mg/ml, successfully reduced the NO level induced by histamine in HUVEC, with a percentage inhibition 51.66 %. However, at two other concentrations, 0.1 and 0.2 mg/ml, AEBO failed to reduce NO levels, and did not have a significant effect on NO levels, compared with the negative control. The association between reductions in NO levels and endothelial hyperpermeability may indicate the mechanism by which AEBO blocked the activation of PLC signalling and subsequently downregulated NO production.

### AEBO abolished histamine-induced cGMP production

**Fig. 5:**
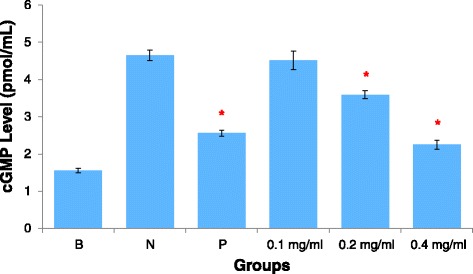
Effect of AEBO on histamine-induced cGMP production in cultured HUVEC. Three independent experiments were performed in triplicate. Data were expressed in mean ± SEM. Data were analyzed by One-way ANOVA. *P < 0.05 significant different from negative control. (B = basal level; N = Treated with histamine only; P = Drug control, 10 μM of LY 83583)

Histamine, at a concentration of 100 μM, increased cGMP production up to three fold compared with basal levels (1.55 ± 0.11 pmol/ml) (Fig. 5). AEBO at 0.1 mg/ml did not have a significant effect on histamine-induced cGMP production. However, 0.2 and 0.4 mg/ml of AEBO notably reduced the levels of cGMP in HUVEC when stimulated with histamine at 3.59 ± 0.11 pmol/ml and 2.25 ± 0.12 pmol/ml, respectively. The maximal inhibitory rate (77.45 %) was found at 0.4 mg/ml of AEBO. Overall, our data suggested that histamine-induced endothelial hyperpermeability was involved in the NO-cGMP signaling pathway; however, endothelial hyperpermeability was abolished by pretreatment of HUVEC with AEBO.

### AEBO suppressed histamine-induced PKC activity in HUVEC

**Fig. 6:**
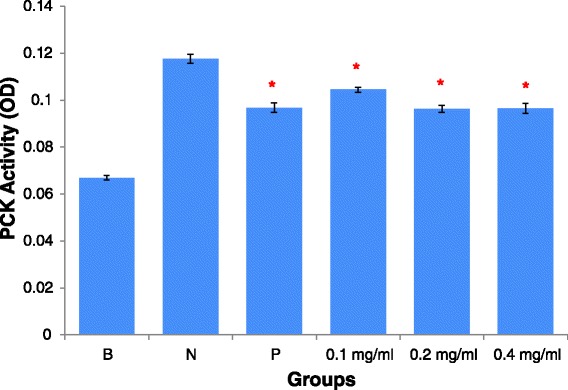
Effect of AEBO on histamine activated PKC activity (OD = optical density) in cultured HUVEC. Three independent experiments were performed in triplicate. Data were expressed in mean ± SEM. Data were analyzed by One-way ANOVA. *P < 0.05 (B = basal level; N = Treated with histamine only; P = Drug control, 10 μM of GF109203X hydrochloride)

We wanted to know whether AEBO exerts its effect on histamine-induced endothelial hyperpermeability through the PKC pathway. To answer this question, the effect of GF109203X hydrochloride, a specific inhibitor of PKC activity, on the levels of PKC protein in histamine-treated HUVEC was measured by ELISA. As shown in Fig. 6, stimulation of histamine led to upregulation of PKC activity in a HUVEC monolayer. All of the AEBO concentrations ranging from 0.1 to 0.4 mg/ml showed significant reductions in PKC activity. The maximal inhibitory rate of 41.62 % was produced by 0.4 mg/ml of AEBO. Moreover, the positive control also showed a significant difference, relative to the negative control, with a percentage inhibition of 41.62 %. These data suggested that histamine-induced endothelial hyperpermeability involved the activation of PKC, which could be suppressed by AEBO.

## Discussion

Previous studies have demonstrated anti-inflammatory and antihistamine activities in aqueous extracts of *B. orellana* (AEBO). These activities were implied by suppression of vascular fluid extravasations induced by histamine in animal model approaches [12]. Thus, extended works were carried out to elucidate its mechanism of action against histamine-induced endothelial hyperpermeability *in vitro*. The present study demonstrates that AEBO exhibits an anti-hyperpermeability effect induced by histamine in human umbilical vein endothelial cells (HUVEC). These activities are closely related to the suppression of PLC-NO-cGMP signaling.

In order to show that AEBO were capable of producing similar results as in an animal setting for the vascular permeability assay, an *in vitro* vascular permeability assay was performed. *In vitro* studies of endothelial permeability that have been used extensively and successfully are reported in the literature [16–18]. The principle of the assay involves the measurement of permeability of an endothelial monolayer, assessed by the amount of dye that leaks into the lower chamber from the upper chamber [16]. This assay is a good system for better understanding the molecular mechanisms of human endothelial permeability. The current study demonstrated that AEBO significantly suppressed histamine-induced endothelial hyperpermeability and this effect was found to be similar to that of the animal model reported previously. This confirms that AEBO exhibited a protective effect on the endothelial barrier.

Next, our study attempted to elucidate the mechanism of action of AEBO on the cellular and molecular levels. Histamine, an endogenous inflammatory mediator, increases endothelial permeability by binding to its receptor, H_1_, a G-protein coupled receptor (GPCR) found on endothelial cells [19]. A conformational change in the GPCR leads to the activation of phospholipase C (PLC), which catalyses the production of inositol triphosphate (IP_3_) and diacylglycerol (DAG) [8, 20]. IP_3_ is then converted to a more stable form, inositol monophosphate (IP_1_). Due to the short of half-life of IP_3_, most assays involve the use of radioisotopes, hence, for safety and health concerns, the determination of IP_1_ levels was chosen in the current study to indicate the activity of PLC. Based on results that were obtained, pretreatment of HUVEC with AEBO significantly reduced histamine-stimulated PLC activity. This may indicate that AEBO reduces endothelial hyperpermeability involved in suppression of PLC activity. However, the effects of AEBO on downstream signaling pathways remain unclear.

Thus, quantification of calcium signaling was performed in the present study. Inositol phosphate, especially IP_3_, is an important second messenger that induces the release of calcium from the endoplasmic reticulum and the subsequent rise in intracellular calcium [21]. Calcium is known to be an important key regulator of a variety of cellular functions, including the maintenance of endothelial cell integrity [22]. However, upregulation of intracellular calcium by inflammatory mediators leads to endothelial barrier dysfunction [23, 24]. In addition, recent findings have suggested that calcium influx through cation channels due to the depletion of intracellular calcium stores plays an important role in increased endothelial permeability [25]. In the current study, calcium signalling assays were performed in both the presence and absence of calcium. However, it appears that AEBO is mainly involved in suppression of intracellular calcium release, rather than extracellular calcium influx. This suggests that AEBO may interfere with the stimulation of membrane-bound enzyme complexes such as PLC, thus reducing the production of by-products, such as IP_3_ and DAG (Chandra and Angle [15]). This eventually, leads to a decrease in intracellular calcium release stimulated by IP_3_.

It is well established that the NO-cGMP signaling cascade plays a key role in the regulation of endothelial permeability [26–28]. Numerous studies have shown that increased endothelial permeability is caused by activation of the NO-cGMP signalling pathway [28, 29]. In contrast, some studies demonstrated a barrier-enhancing effect of cGMP [30, 31]. Different effects of NO and cGMP on endothelial cells have been reported; this is likely due to differences in experimental settings, such as the species used, the particular vascular bed and the physiological condition of the cells [32]. While upregulation of NO-cGMP signalling induced by histamine in HUVEC was showed in the current study, this condition was abolished by preincubation with AEBO. Thus, the current findings show that AEBO is capable of downregulating the NO-cGMP signaling pathway induced by histamine in HUVEC.

Protein kinase C (PKC) activity is known to be activated by diacylglycerol (DAG), an end product of the catalytic action of PLC [33]. In addition, Ca^2+^ signaling pathways also enhance PKC activity [34]. As activation of PKC is involved in the phosphorylation of cytoskeletal protein as well as in the reorganization of intercellular junctions, it plays an important role in the regulation of endothelial permeability [35]. Indeed, upregulation of PKC activity in endothelial cells was shown to increase endothelial cell permeability [36–38]. However, evidence also showed that the inhibition of PKC activity led to a reduction in the effect of an agonist on hyperpermeability [39]. The current study showed that pre-treatment of AEBO on HUVEC slightly ameliorated the PKC activity induced by an agonist. This suggests that AEBO may be able to interfere with calcium signaling, but fails to alter DAG signalling. However, further research is needed in support of this hypothesis.

## Conclusion

In conclusion, it was suggest that AEBO suppresses PLC activity, which in turn leads to suppression of its downstream signalling pathway. This causes a reduction in calcium concentration, nitric oxide and cGMP production in cultured HUVEC when induced by histamine. Inhibition of the PLC-NO-cGMP signalling pathway and PKC activity by AEBO contributes to the suppression of the increase in endothelial permeability, which may lead to complicated illnesses.
